# Communicating international politics narratives of security, democracy and human rights in contemporary society: indexing and analysis using online monitoring data

**DOI:** 10.3389/fsoc.2024.1441787

**Published:** 2024-10-17

**Authors:** Anca Parmena Olimid, Cătălina Maria Georgescu, Cosmin Lucian Gherghe

**Affiliations:** Political Sciences Specialization, Faculty of Social Sciences, University of Craiova, Craiova, Romania

**Keywords:** security, democracy, human rights, society, concept

## Abstract

**Objectives:**

The role of the current research is to set up a conceptual and analytical framework of the security, democracy and human rights linkage in contemporary society mainly based on the indexing, monitoring and research of online content for the period 1945–2019.

**Methods and methodology:**

Due to the focused timeline outlining the scientific research for the period 1945–2019, the paper favors both quantitative and qualitative approaches that follow a systemic framework of several thematic clusters which act as supporting pillars of analytical research by using Google Ngrams: (a) norms and constitutional values and principles; (b) civil and legal liberties; (c) human and individual approaches; (d) institutional governance and organizational establishment; (e) freedoms, civil and political rights.

**Results and discussions:**

The research results explore the variations in the frequency of appearances of the selected concepts in the specialized literature indexed by the Google platform indicating essential aspects of the conceptual and theoretical evolutions in strict dependence to the significant resolutions of the Security Council of the United Nations adopted in the same period and focusing on three central concepts: security, democracy and human rights. The spectrum of research singles out fifteen figures and an integrated analysis of contextual, historical, political, institutional, legal and social factors.

**Conclusion:**

The research aims to expand the field of reference and analysis of the three notions of security, democracy and human rights by integrating the multifactorial and multi-conditional analysis for the interpretation of the results of the fifteen figures. In the broader framework of online monitoring and complementarily with the spectrum of UNSC resolutions, the research will use contemporary topics, intensely debated, used and monitored, focusing on the conceptual and linguistic study and on the relationship between the analytical inventory of scientific research and the international decision-making spectrum. This relationship is mediated by the lexicon of politics and the sociology of international relations, which reflected a growing evolutionary linguistic semantics since 1945, the year of the establishment of the United Nations.

## 1 Introduction

The study of the conceptual and analytical evolution of the three concepts (security, democracy and human rights) requires a multi-conditional and multivariate assessment (Besson, 2011; Broberg and Sano, 2018; Brunkhorst, 2009) of the contextual, structural and functional factors of configuration of state, society, governance, civil, political and social rights, social status, participation, involvement and civic commitment (Gafuri, 2022; Goodhart, 2008).

The five configurations specified above have been explored and investigated with multiple interpretations in the scientific literature for the period 1945–2019 by focusing on a plurality of social factors, historical and political events and legal causalities (Quintavalla and Heine, 2019; Usatin and Fauriol, 2022; Zysset and Çalı, 2023).

The evolution of the social and political space at the global level registered new trends and perspectives after the establishment of the United Nations (UN) in San Francisco in 1945. For the past eight decades, the resolutions of the Security Council (SCRs) of the United Nations represented and highlighted the need to respect the principles of the Charter of the United Nations (1945), but especially the guarantee of the social and political rights of both the individual and the state.

In a first sense, the research generates a receptive feedback mechanism, because the documentary analysis of the resolutions adopted by the UNSC highlights both legal solutions to the various case studies presented, but especially the way in which the legislative technique of drafting the resolutions registers new concepts, associations of words or phrases.

We will therefore highlight evolutionary stages in the field of conceptualization but also the discursive technique in the fields of politics and sociology of international relations.

In a second sense, the analysis of the frequency of occurrence of selected concepts and word associations using the Ngram method will generate a double validation and argumentation mechanism at the social and functional level. At the social level, the results of the Ngram analysis will highlight and demonstrate how the scientific literature has taken over, used and employed the different selected concepts.

At the functional level, the scientific literature has progressed and employed new directions of conceptual evolutions, appreciating the emergence of new social and political actors, new mechanisms and economic policies, but also a new register of norms and principles in the legal sphere. The study of the three concepts security, democracy and human rights involves the exploration of a complex framework of interpretation, synthesis and analysis of more than eight decades of specialized scientific literature.

A first perspective considers the research associated with the international political system involving the functionality of political society and the role of institutions and resources.

The second perspective explores the most frequent associations of concepts in the context of the functionality of the UNSC after 1945. In order for this research to be relevant, we have inventoried in the fifteen figures the linguistic associations and extra-linguistic conceptualizations compatible and representative to the decisional and contextual communicative space of the UNSC in the period of 1945–2019 (armed conflicts, ceasefire, peace process, commitment and international support, post-conflict decisions, resolutions for the peaceful resolution of conflicts, security and migration, resources and good governance).

Furthermore, the analysis vectors of the present research investigate the multiple causalities rigorously employing a cluster research and exploration framework based on the synthetic analysis of a complex of perspectives:

- the perspective of the individual, of the citizen in the democracy-human rights-civil society relationship (Hill, 2024; Jones, 2021; Poppe and Wolff, 2017; Poppe et al., 2019; Rothstein, 2019) delineating the enhanced perspectives for women and children (Martín de la Rosa and Lázaro, 2019; Heathcote, 2018; Jansson and Eduards, 2016). Researches on human rights advances have outlined the use of “human dignity” with a universal scope and contextual relatedness (Kane et al., 2024).
- the perspective of social governance, societal security, participation and the institutionalized establishment (Bradby et al., 2020) investigating the perspective of human security (Gilder, 2021) and also UN competences, authority and practices in establishing security matters (Barber, 2021). Research on content analysis has shown a dynamic transformation and conceptual evolution in political discourse (Dib and Sandy, 2023).
- the perspective of human rights and norms according to the principles of democratic governance and social action agenda (Dandashly, 2018; Achermann and Besson, 2023; Ekström and Stevanovic, 2023). Most recent literature has deepened conversations on security and human rights especially related to political, social and economic aspects (Bonadiman and Soirila, 2019).
- the doctrinal and interpretative perspective of configuring the pillars of the democracy-human rights relationship as defined above (Landman et al., 2012; McAuliffe, 2022). There have also been extensive researches over the influences or even shifts in policy legitimation and discourse through different narratives of national security (Sandford, 2024).

Therefore, the research objectives focus on analyzing the nexus between security, democracy and human rights proposing, first of all, a thorough evaluation of the governing spectrum and the relevant international documentation from the period 1945–2019, but also a detailed complementary, convergent and associated core screening of the scientific literature published in the same period.

In a first perspective, such an approach traces a correlated interaction absolutely necessary for understanding the mechanisms of global governance in contemporary society, but also for following the evolutionary-discursive trajectory of international politics in the last seven decades.

A second perspective extensively debates the spectrum of scientific literature and articulates the aspects of institutional commitment, but also the consolidated mechanisms of interaction with the public space.

Also, the two perspectives explore dynamically and progress a complex of subjects related to the monitoring of international law, but also the systematic approach of the international society starting with the year 1945 until the year 2019.

## 2 Materials and methods

### 2.1 Research methodology and data collection

By configuring the pillars of analysis and centering the three dimensions of investigation: security, democracy and human rights, the methodology prospects the quantitative and qualitative analysis of the scientific content indexed by the Google platform starting from 1945 till 2019.

Google Ngram Viewer is based on an online search engine that records the frequency of occurrence of a word or string of words (n-gram) found in the scientific literature indexed in the period 1945–2019 in the content digitally indexed by Google for this period.

The period selected for the analysis 1945-present takes into account the challenging context generated by the often antagonistic and contrasting positions and interests of the member states, this research aims to initiate an innovative research on the evolution of emerging concepts and word associations in the sphere of politics and sociology international relations engaging a bi-focused analysis at the analytical and linguistic level. The data were retrieved and collected in the period May 02–May 25, 2024.

At the analytical level, the investigation of the emergence and evolution of the main conceptual trends in the resolutions of the UN Security Council (UNSC) adopted from June 1945 and present.

### 2.2 Emerging conceptual trends and thematic modeling

At the linguistic level, we consider the analysis of the frequency of appearance of the selected terms in the scientific literature from the period 1945–2019 indexed and listed by the Google Books platform. The N-gram technology involves the use of the multilingual context provided by the corpus of texts indexed by Google books.

In the coding process, the n-gram contribute to the contextualization of international politics, developing and employing mechanisms for an in-depth understanding of the narrative nexus centred on the relationship between security, democracy and human rights in contemporary society.

Second, the word sequences selected for analysis validate an integrated analysis model characterized by:

- transparency and accuracy of the documentation selected for analysis (twenty-five UNSC resolutions relevant to the focal theme of the research);
- the reliability of the research results that help to extract the relevant contexts and periods for the evolution of international politics.

For the context of the discussion generated by the research theme, selected ngrams refer to words or sequences of words (n-gram as a representation of a sequence of *n* words), as follows:

- if *n* = 1, n-gram is called unigram (one word associated with research and analysis);
- if *n* = 2, n-gram is called bigram (2-gram): a joining of two connected and associated words;
- if *n* = 3, the n-gram is called a trigram (3-gram): a sequence of three words and.
- if *n* = 4, n-gram is called fourgram (4-gram) which associates four words in a single associated sequence.

The selection of concepts and word associations (n-gram) was made manually on the basis of SCRs considering the evolution of the international plan and the focused and accentuated movement towards seven themes of the syntax ordered by the legislative technique of the resolutions in the last eight decades. Thus, the first three themes are static approaches identifiable in an interdisciplinary context of the humanitarian assistance which represent the systemic prototype of the security-democracy-human rights relationship in the analysis of contemporary society. The second set of themes instruments the generic dialogue between the individual and the institutional plan, its fundamental scientific contribution being centered on the morpho-lexical processing of four concepts standardized in the UNSC resolutions: humanitarian, protection, governance and reform.

### 2.3 Data analysis and data exploration

The limits of the research are generated by the normalization of the indexed corpus for a specific year, at a relative level with the number of books published in the same year. Another limitation involves the sphere of the overabundance of scientific literature from the last eight decades, considering the emergence of some terms and the rhythm of their use in natural language.

**Figure 1:**
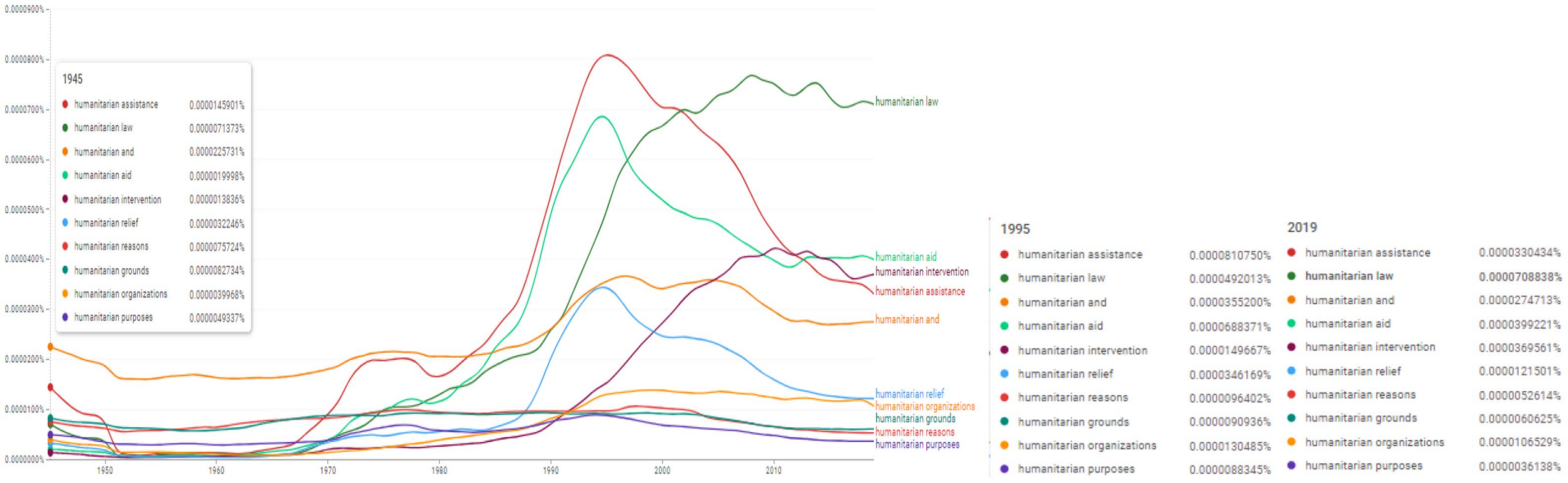
Ngram wildcard search: “humanitarian*.” Source: search results in Google Ngram Viewer.

**Figure 2:**
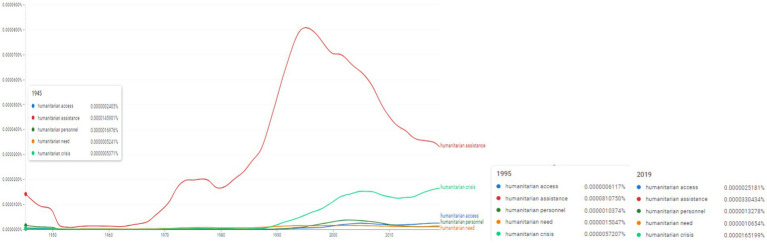
Ngram search: selected association to the concept “humanitarian.” Source: search results in Google Ngram Viewer.

**Figure 3:**
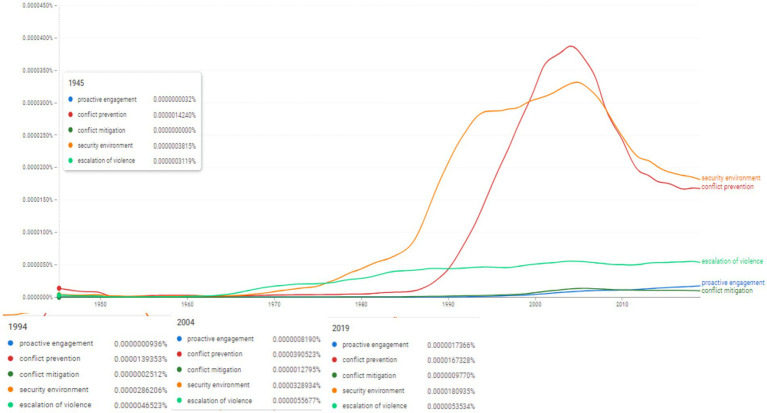
Ngram search: selected associations on prevention and resolution of conflict. Source: search results in Google Ngram Viewer.

**Figure 4:**
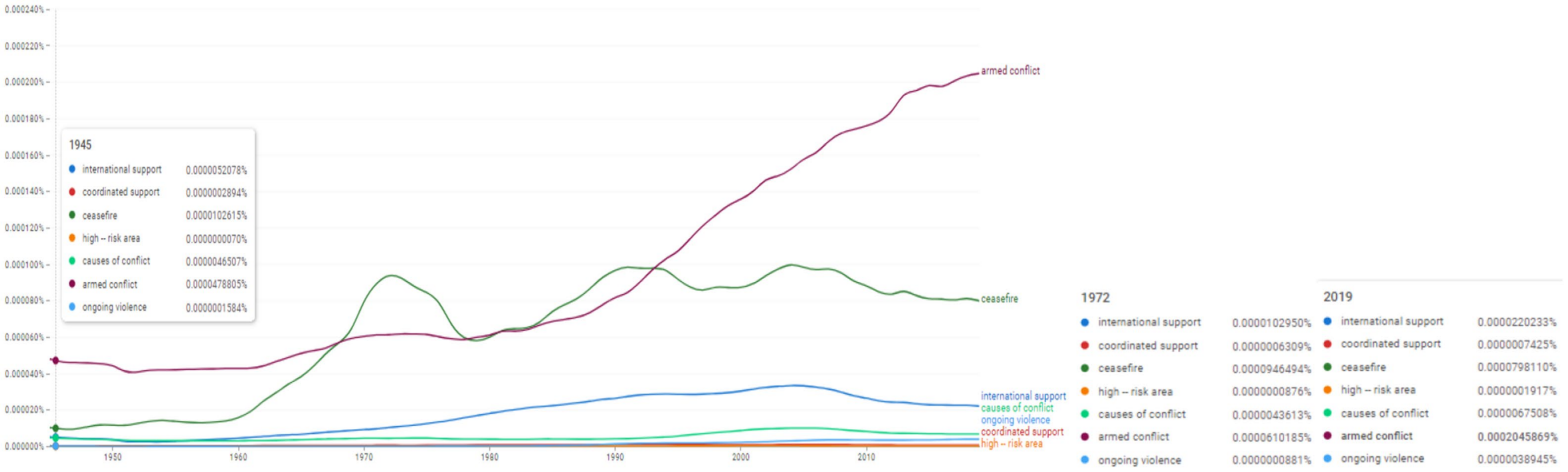
Ngram search for selected conceptual associations on conflict proliferation and internationaĺ action. Source: search results in Google Ngram Viewer.

**Figure 5:**
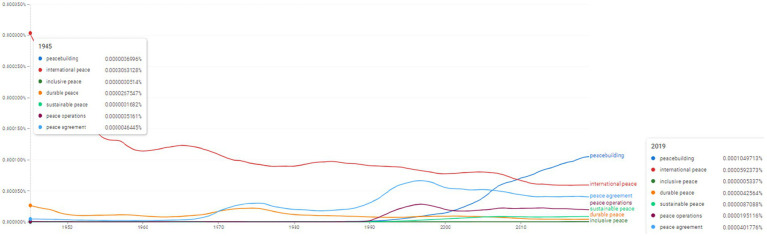
Ngram search: selected concepts associated to conflict development process and actors. Source: Search results in Google Ngram Viewer.

**Figure 6:**
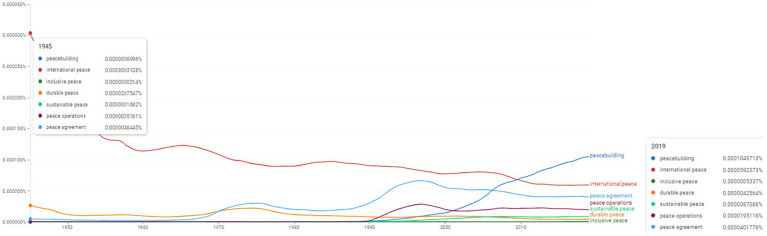
Ngram search: conceptual associations around the concept of “peace”. Source: search results in Google Ngram Viewer.

**Figure 7:**
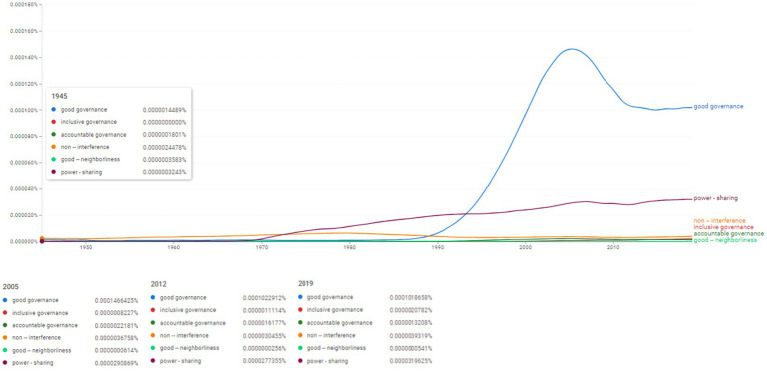
Ngram search: conceptual associations within the sustainable governance thematic area. Source: search results in Google Ngram Viewer.

**Figure 8:**
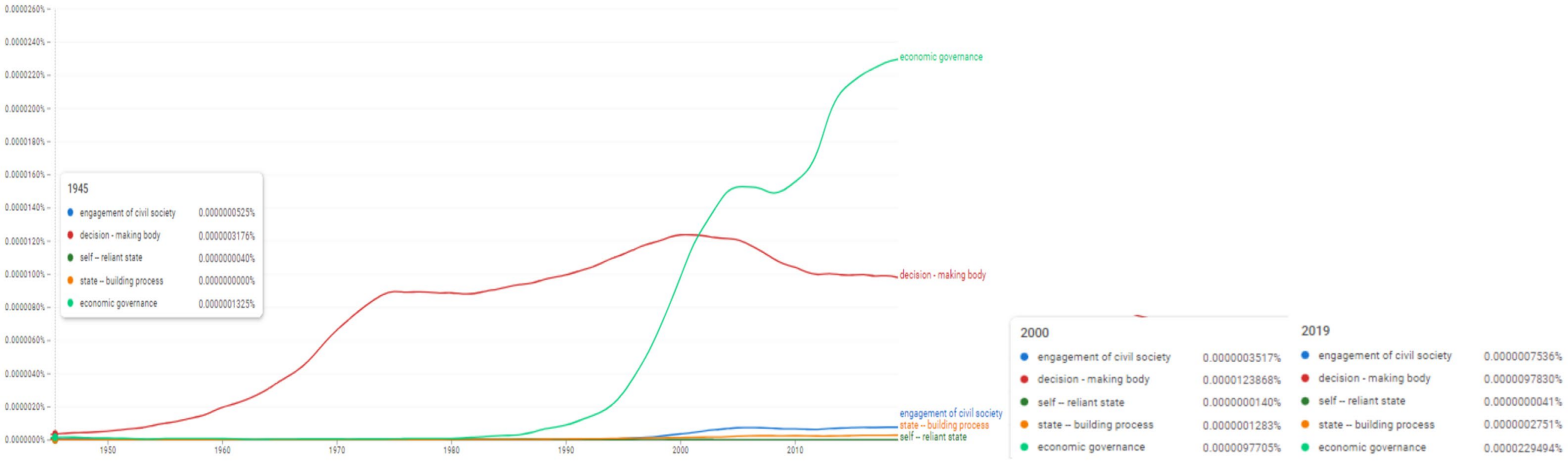
Ngram search: State-society related conceptual associations. Source: search results in Google Ngram Viewer.

**Figure 9:**
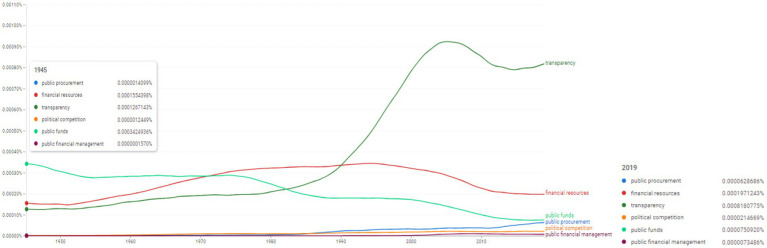
Ngram search: financial mechanisms related conceptual associations. Source: search results in Google Ngram Viewer.

**Figure 10:**
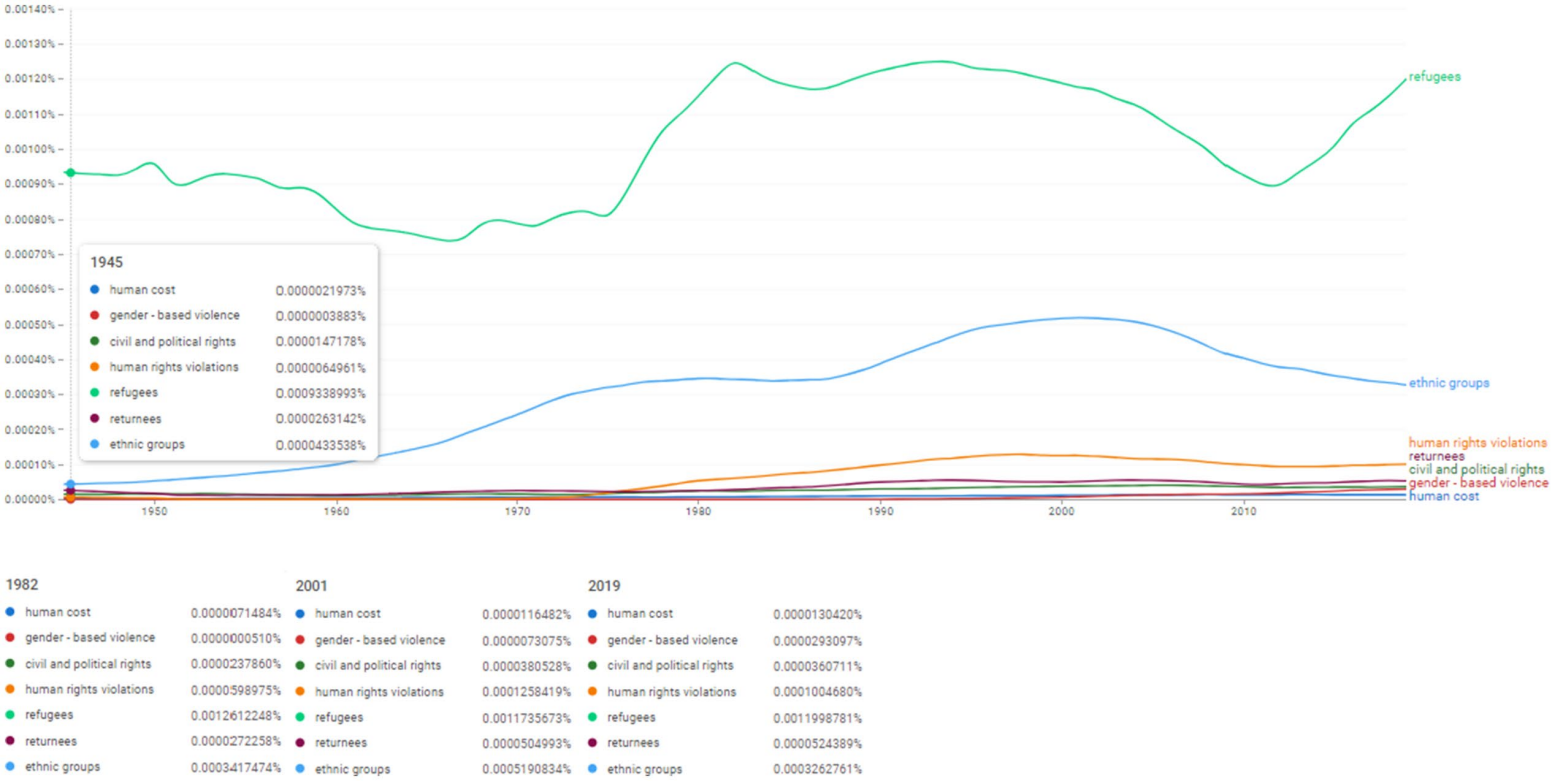
Ngram search: human cost and human rights associated concepts. Source: search results in Google Ngram Viewer.

**Figure 11:**
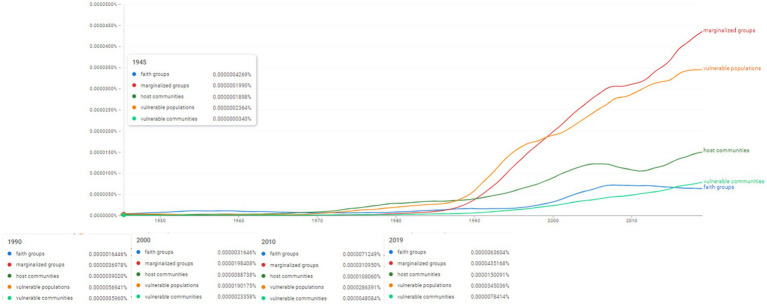
Ngram search: vulnerable populations and marginalized groups associated concepts. Source: search results in Google Ngram Viewer.

**Figure 12:**
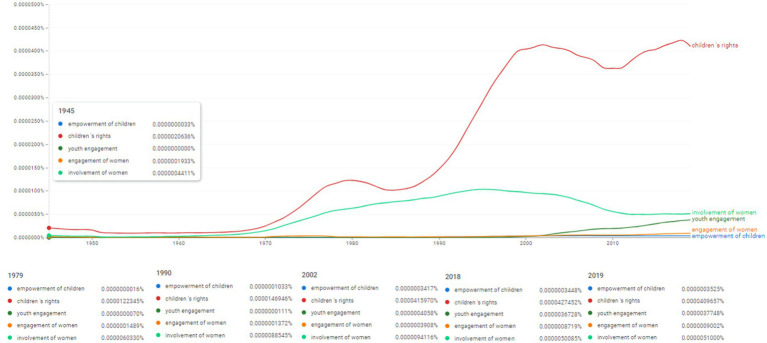
Ngram search: empowerment of women and children associated concepts. Source: search results in Google Ngram Viewer.

**Figure 13:**
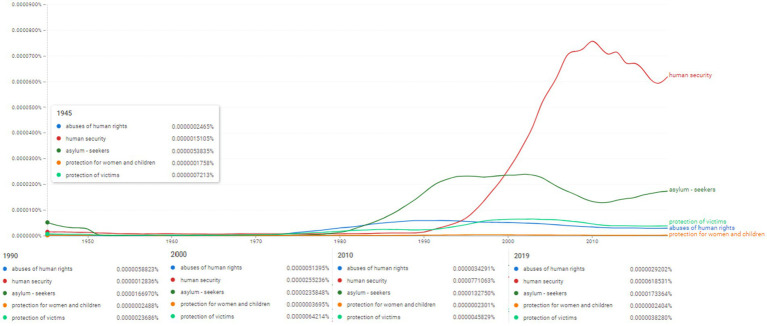
Ngram search: human security and protection for women and children associated concepts. Source: search results in Google Ngram Viewer.

**Figure 14:**
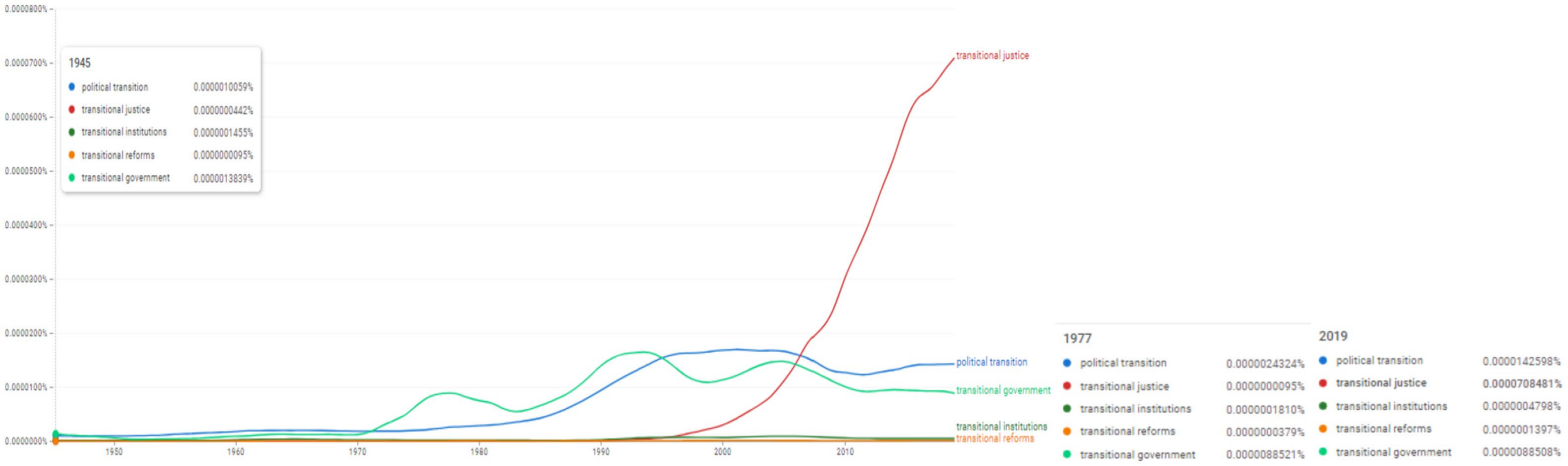
Ngram search: political transition and transitional justice. Source: search results in Google Ngram Viewer.

**Figure 15:**
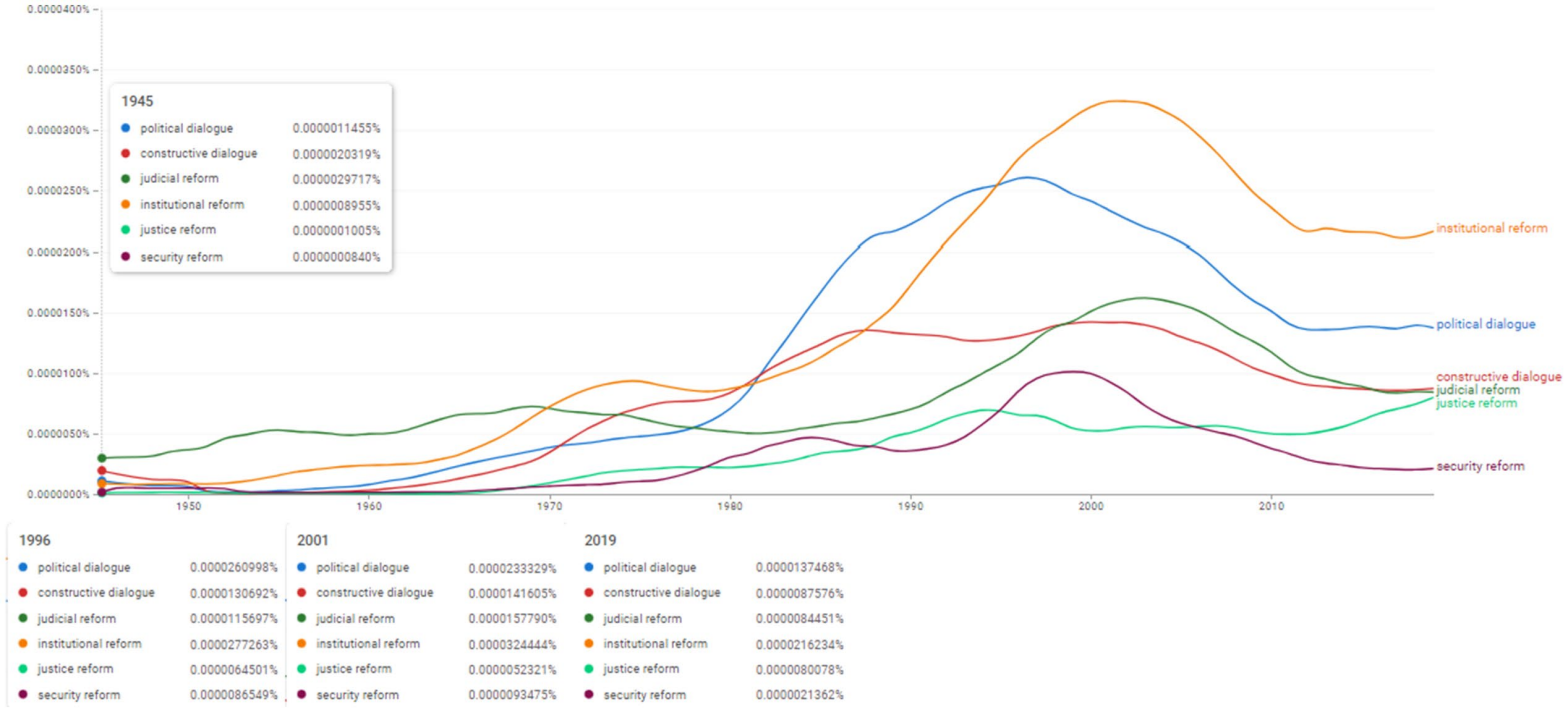
Ngram search: political dialogue and reform.

In this context, the research follows a four steps framework considering the following aspects: (1) the analysis uses the Ngram method by identifying the conceptual frequency variations of the selected topics; (2) the topics are selected manually in order to investigate a variety of connections, associations and linguistic phrases used by the scientific literature; (3) the researched period is very extensive starting from the year 1945 until 2019 employing several topics of research and analysis and four pillars of analysis with certain topics in each pillar of the research, as follows: (i) humanitarian causes and social issues (Figures 1–3); (ii) peace, security and defense (Figures 4–6); (iii) state-building process and governance (Figures 7–9); (iv) social reforms, liberties and political rights of vulnerable groups (Figures 10–13); (v) public diplomacy and leadership (Figures 14, 15).

Methodologically, the research was structured to answer a series of questions:

Q1: How and when did conceptual trends related to security, democracy and human rights appear and evolve in the resolutions of the UN Security Council (UNSC) during the selected analysis period of 1945–2019?

Q2: How did the security-democracy-human rights relationship evolve in the scientific literature?

Q3: What is the frequency of appearance in the online indexed scientific literature from the period 1945–2019 of selected concepts and word associations (n-grams) specific to the security-democracy-human rights relationship?

Q4: Is there a correlation between UN Security Council (UNSC) resolutions and the frequency of appearance of concepts in the scientific literature during 1945–2019?

Q5: Do concepts and terminology operationalized in the UN Security Council (UNSC) resolutions during a certain period indicate a central theme in the scientific discourse?

Q6: Do selected concepts and terminology coin new types of approaches in the decision-making and implementation process of the UN Security Council (UNSC) resolutions?

### 2.4 The correlation of the frequencies in the scientific literature with the linguistic resources of the UNSC resolutions

For the discussion section, we correlated the results of the analysis of the frequency of concepts and associations of concepts for the period 1945–2019 with the linguistic framework of the twenty-five supporting resolutions adopted by the UNSC in the same period.

The concepts selected for the analysis are synthesized in a standardized structure according to the theme selected in the analysis section to explain the fundamental reference of the scientific literature processed and indexed by Google books (corpus of written, printed texts, monographs, reports, electronic dictionaries, other associated thesaurus lexical or conceptual or terminological content) with the decision-making framework of the UNSC. This correlation also has the character of synthesis and recognition of the importance of the SCRs in the period 1945–2019 for the scientific literature in the field from the same period.

The compatibility and analysis of representation and correlation between the decision-making framework of the UNSC and the framework of representation of scientific literature becomes indispensable for the very functionality of the international political scene, being a fundamental reference for the analysis and social functionality of the global space after the Second World War as that the pressure exerted by the implementation of a status-quo of peace, solidarity and dialogue is increasing.

## 3 Results

The research results explore the variations in the frequency of appearances of the selected concepts in the specialized literature indexed with the help of the Google platform indicating essential aspects of the conceptual and theoretical evolutions in strict dependence with the significant moments of the evolution of the political and legislative history of the three central concepts: security, democracy and human rights for the period between 1945 and 2019. The spectrum of research singles out fifteen figures and an integrated analysis of contextual, historical, political, institutional, legal and social factors.

With the purpose of identifying the most frequent associations with the concept “humanitarian” we operated a wildcard search on Google Ngram Viewer.

During 1994–1995 the following concepts were trending: “humanitarian assistance” (0.0000810750% versus 0.0000330434% in 2019), “humanitarian aid” (0.0000688371% in 1995 versus 0.0000399221% in 2019) and “humanitarian relief” (0.0000346169% in 1995 as opposed to 0.0000121501% in 2019). The concept of “humanitarian law” reached its peak in 2008 (0.0000774205% in 2008 versus 0.0000708838% in 2019), and “humanitarian intervention” in 2010 (it reached 0.0000426758% versus 0.0000149667% in 1995 and 0.0000369561% in 2019). Three concepts experienced a similar trend: “humanitarian grounds,” “humanitarian reasons” and “humanitarian purposes” (Figure 1). All three concepts share a decrease in usage between 1950–1970, followed by an increase in frequency especially throughout the 1990s: in 1995 the concept of “humanitarian grounds” was used with a rate of 0.0000090936%, “humanitarian reasons” reached 0.0000096402% and “humanitarian purposes” 0.0000088345%. Since the middle of 2000s the values for all three concepts almost halved towards 2019 (listing 0.0000060625, 0.0000052614 and 0.0000036138%, respectively). The concept of “humanitarian organizations” shared a similar trajectory. The frequency values dropped after 1950 and began to rise after 1970. In 1995 “humanitarian organizations” reached 0.0000130485% (thrice the value registered in 1945 of 0.0000039968%) and since then mantained the relative frequency. In 2019 it scored 0.0000106529%.

The results obtained using the wildcard search in Figure 1 (e.g., “humanitarian assistance” which scored 0.0000810750% in 1995) were compared to the selected terms “humanitarian access,” “humanitarian assistance,” “humanitarian personnel,” “humanitarian need” and “humanitarian crisis” (Figure 2). During the period 1950–1990 these latter concepts of “humanitarian access,” “humanitarian personnel,” “humanitarian need” and “humanitarian crisis” experience a limited usage, however, following 1990 the terms begin to appear extensively in ngrams, suggested a widening usage (for instance, “humanitarian crisis” scores 0.0000165199% in 2019). In order to have a comparable value, one has to account that the concept “humanitarian” reaches its peak in 2004 with the value of 0.0007908786%.

Further we aimed at identifying the frequencies of usage for certain conceptual associations around security issues and conflict escalation by employing Ngram search for the following concepts: “proactive engagement,” “conflict prevention,” “conflict mitigation,” “security environment,” “escalation of violence” (Figure 3). We observe a very limited usage during the 1950s and 1960s. At the beginning of the 1970s the term “security environment” witnesses a rapid increase in usage reaching 0.0000328934%, it falls towards 0.0000180935% in 2019. “Conflict prevention” witnesses its rise in the late 1980s, it reaches its peak in 2004 (0.0000390523%), and shows a similar downward trend towards 2019 when it scores 0.0000167328%. A usefull insight for comparison is identifying the peak in the usage of the concept “conflict” in 2005 at the value of 0.006371189%.

Consequently, the results on the frequence of ngrams show an evolving research interest on a human rights and democracy approach over prevention and resolution of conflicts (Besson, 2011).

Within the ngram search performed around conflict proliferation and international action theme concepts, we emplyed the following concepts: “international support,” “coordinated support,” “ceasefire,” “high-risk area,” “causes of conflict,” “armed conflict,” “ongoing violence” (Figure 4). Among these, “armed conflict” appears to have increased its frequency especially after the beginning of the 1990s. In 2019 it reaches its peak at the value of 0.0002045869%. Another interesting evolution appeared for the concept of “ceasefire” which seems to have been used more frequently after 1960. In 1972 it reaches 0.0000946494%, comparable to the values in 2019 of 0.000079811%. A useful term for comparison is that of “proliferation” which registered its peak in 1994 at the value of 0.0010920696%.

The results are consistent with the literature which shows a deeper interest in the evolution of international coopertation (Achermann and Besson, 2023) and the international organizations competence in legal matters and international action (Barber, 2021).

For the next Ngram search we selected concepts associated to conflict development process and actors: “early-warning system,” “international community,” “protection of civilians,” “post-conflict situation,” “conflict resolution” (Figure 5). The concept of “international community” appears to be used most frequently during the period starting with 1997 (0.0004261663%) and reaches a peak in 2003 (with the value of 0.000431658%). After that period, its usage seems to have decreased until 0.0002691158% in 2019. Another interesting evolution is that of the concept of “conflict resolution” whose frequency increases after 1960, especially towards the late 1990s: in 1997 its frequency reaches 0.0001699754% and reaches a peak in 2003 at 0.000174648%. “Protection of civilians,” however, seems to be used more frequently after 2000 (in 2003 the value reaches 0.000004848%, while in 2019 it registers 0.0000121832%). Consequently, the literature on the international community as an actor in conflict resolution and UN Security Council responsibilities in the evolution of discourse has grown (Byers, 2020).

The concept of “peace” appears to be used in many instances in the scientific literature. Equally interesting is its evolution dynamics. While “international peace” sees a relative decline (from the values of 0.0003063128% identified for 1945 towards 0.0000592373% in 2019), other concepts experience a growth in usage: “peacebuilding,” “inclusive peace,” “durable peace,” “sustainable peace,” “peace operations,” “peace agreement” (Figure 6). For instance, the concept of “peacebuilding” appears to be used more frequently after 1990. In 2019 it reaches 0.0001049713%. An equally interesting evolution is seen with the concept of “peace agreement” which appears to have been used scarcely until 1970. After that period, the concept seems to be used more intensely, as it reaches 0.0000401776% in 2019. In order to benchmark the frequencies, in 1945 the concept of “peace” scored 0.0111148039%, while in 2019 it reaches 0.0068837933%.

To ilustrate the sustainable governance thematic area we performed an Ngram search for the following concept frequencies: “good governance,” “inclusive governance,” “accountable governance,” “non – interference,” “good – neighborliness,” “power-sharing” (Figure 7). The most intense frequency in this category was registered for the concept of “good governance” which indicate that the concept began to be used keenly after 1990; from the value of 0.0000014489% in 1945 it rose to 0.0001466425% in 2005 and to 0.0001018658%. This might be compared to the use of the concept of “governance” which similarly rose after 1990 in order to reach its peak in 2019 at the value of 0.0027573034%. A constant rise in frequency values is seen in relation to the concept of “power-sharing” which seems to be used after 1970 and reaches its highest frequency in 2019 (0.0000319625%).

Moreover, we targeted the following selective state-society related conceptual associations: “engagement of civil society,” “decision-making body,” “self—reliant state,” “state—building process,” “economic governance” (Figure 8). Among those concepts, “economic governance” appears with incresed frequency beginning with 1990, in 2000 it reaches 0.0000097705%, while in 2019 it reaches its highest frequency at 0.0000229494%. The concept of “decision-making body” experienced a rise in frequency after 1945. In 2000 it reaches 0.0000123868%, while in 2019 it was used with a frequency of 0.000009783% indicating the increased tendency to be used in the sceintific literature. “Self-reliant state” appears in 1912 with the value 0.0000000287%. It then reaches 0.0000000140% in 2000 and 0.0000000041% in 2019. However, we observe that the concept of “engagement of civil society” began to be used later beginning with 2000 and mantained its trend towards 2019.

The next Ngram search aimed at identifying the frequencies of concepts related to financial mechanisms: “financial resources,” “transparency,” “public procurement,” “political competition,” “public funds,” “public financial management” (Figure 9). Among these concepts, the issue of transparency seems to be of impact as indicated by the increase in frequency values since 1945 (0.0001267143%) until 2019 (0.0008180775%). The concept of “financial resources” seems to have been used with a similar intensity in 2019 (0.0001971243%) as it was used back in 1945 (0.0001554398%). We have to comment on the issue of “public procurement” conceptual usage which increased especially after 2013 reaching the value of 0.0000628686% in 2019.

Aiming at identifying the frequencies of usage for human cost and human rights associated concepts, the Ngram search focused on the following concepts: “human cost,” “gender-based violence,” “civil and political rights,” “human rights violations,” “refugees,” “returnees” and “ethnic groups” (Figure 10).

Throughout the analysed period, the concept of refugees was used with different intensities; the Ngram values registered for 1945 show 0.0009338993%. Beginning with the late 1970s, the values rise and reach a peak in 1982 at 0.0012612248%. The term remains intensively used throughout the 1990s and 2000s (for instance, in 2001 the registered frequency was 0.0011735673%). However, we observe a slight decrease in usage after mid 2000s. The values change their downward trajectory and in 2019 the frequency of the term “refugees” reaches a new peak of 0.0011998781%. Likewise, the concept of “returnees” has been used with the same frequency since 1945 (0.0000263142%). Throughout the 1980s the frequency started to grow (in 1982 the value was 0.0000272258%). In 2001 the value was already 0.0000504993% and remain aroundthis value until 2019 (0.0000524389%).

The concept of “civil and political rights” registered a frequency of 0.0000147178% in 1945. After 1970 it began to be used more frequently, almost doubling its usage to 0.0000237860%. By 2001 it reached 0.0000380528% and maintained the rate of this frequency until the end of the analysed period.

One of the most interesting situations is that of the concept “ethnic groups.” In 1945 it was used with a frequency of 0.0000433538%. After 1950 it seems to be used more often, its frequency values rising constantly. In 1982 it was used with a frequency of 0.0003417474%, while in 2001 its frequency almost doubled as it reached 0.0005190834%. In the second half of the 2000s the usage seems to slightly decrease, reaching the value of 0.0003262761% in 2019.

The two concepts of “human cost” and “human rights violations” both share a similar rising tendency after 1970. The rise in frequency is especially obvious for the concept of “human rights violations” which reaches 0.0000598975% in 1982 and a peak of 0.0001258419% in 2001 and maintains this rate by 2019 (0.000100468%). We also notice the development of the concept of “gender-based violence” which registered the frequency of 0.0000003883% in 1945 then dropped after 1950. In 1982 the frequency was still low (0.000000051%). It began to rise in the 1990s. In 2001 its frequency in the literature was already 0.0000073075%. The highest frequency in the analysed period is registered in 2019 (0.000029309%). For comparison, the concept of “gender” seems to be used with an increased intensity since the middle of the 1970s. In 1980 it was already used with a frequency of 0.0004230029% which increased greatly until its height in 2019 (0.0053430066%).

In this sense, Jansson and Eduards (2016) offer a critical insight of UN Security Council resolutions on “women, peace and security,” “war regulations,” violence against women during armed conflicts and the gender-security relation. The literature on “securitization” and gender relations in conflict areas has widened with the adoption of UN Security Council resolutions.

The next Ngram search concentrated on identifying the frequencies of vulnerable populations and marginalized groups associated concepts, thus focusing on the following concepts: “faith groups,” “marginalized groups,” “host communities,” “vulnerable populations,” “vulnerable communities” (Figure 11). Though present in the scientific literature, their frequency is weak. This situation changes dramatically especially after 1990s and throughout the 2000s. The concept of “faith groups” is used with increased frequency from 1990 to 2000 and then 2010 (0.0000071249%). The concept of “vulnerable communities” seems to be used more frequently after 1990 (in 2019 it reaches 0.0000078414%). The use of the concept of “host communities” follows the same timeline, increasing its presence in the literature since the 1970s and mostly after 1990. In 2019 the frequency scored 0.0000150091%.

Still, the highest frequency was recorded in the case of two concepts of “vulnerable populations” and “marginalized groups.” “Vulnerable populations” appears to be used more intensely earlier since the 1970s. However, its frequency rises incrementally after 1990 with a continuous growth throughout the 2000s. In 2019 its highest frequency value was 0.0000345036%. The concept of “marginalized groups” appears to have a later application, still after 1990 it shows the same dramatic increase in frequency which establishes a constant growth throughout the analyzed period until its peak in 2019 (0.0000435168%).

The next stage of research aimed at identifying the concepts associated to the idea of empowerment of women and children, thus the Ngram search employed the terms “empowerment of children,” “children’s rights,” “youth engagement,” “engagement of women,” “involvement of women” (Figure 12). We thus notice the tendency to talk about “involvement of women” with a higher intensity than of “engagement of women” (for instance, in 2019 “engagement of women” was used with a rate of 0.0000009002%, while “involvement of women” was used with a frequency of 0.0000051%). The concepts of “youth engagement” and “empowerment of children” appear to be used firstly in 1979, however, in 2019 their frequency reaches 0.0000037748% for “youth engagement” and 0.0000003525%, respectively. Furthermore, the concept of “children’s rights,” though present in the literature, seems to be used more intensely after 1970, with an increased dynamics after 1990. It reaches a peak in 2018 at a frequency of 0.0000427452%.

Continuing the Ngram search for human security and protection for women and children associated concepts, the analysis engaged the concepts of “abuses of human rights,” “human security,” “asylum-seekers,” “protection for women and children,” “protection of victims” (Figure 13). Among all these concepts we have to notice the evolution in the use of “human security” whose frequency increased 20 times between 1990 and 2000, and 60 times between 1990 and its peak of 2010. After this period we observe a slight drop in term usage, however, in 2019, the value rises again to 0.0000618531%. The term was used scarcely during 1950–1980. However, by the middle of 1990s it almost reached its highest frequency (in 2000 it scored 0.0000235848%).

Furthermore, the next stept of the analysis focuses on political transition and transitional justice, consequently the Ngram search was developed using the concepts of “political transition,” “transitional justice,” “transitional institutions,” “transitional reforms,” “transitional government” (Figure 14). Among these terms, “transitional justice” has developped at the middle of the 1990s, and since then it was used intensely; in 2019 its frequency was 0.0000708481%. The term “transitional government” appears to be used after 1970 more intensely (in 2019 its frequency was 0.0000088508%); likewise, the term “political transition” increases its frequency at the middle of the 1980s (in 2019 its frequency was 0.0000142598%).

The final stage of Ngram search focused on political dialogue and reform. Thus the terms selected from this register were “political dialogue,” “constructive dialogue,” “judicial reform,” “institutional reform,” “justice reform,” “security reform” (Figure 15). Although in 1945 the concept of “constructive dialogue” was used with almost double frequency than that of “political dialogue” (0.0000020319% versus 0.0000011455%) the latter sooner increased its usage so that in 1996 the ratio was reversed and the frequency of “political dialogue” (0.0000260998%) doubled that of “constructive dialogue” (0.0000130692%). Since 1945, the concept of “institutional reform” had a gradual growth leading to its peak in 2001 (0.0000324444%). Since 1945 and until 1970 the concept of “judicial reform” had the highest frequency from this category (0.0000029717% in 1945). Since 1970 the frequency values scored higher for the term “institutional reform.” The terms “justice reform” and “security reform” had the lowest frequencies from this category in 1945 and throughout the analysed period. “Security reform” scored highest in 2000 (0.0000093475%), while “justice reform” continued its ascending path towards 2019 (0.0000080078%).

## 4 Discussion

In the process of analyzing and correlating the frequency of appearance of concepts in the scientific literature from 1945–2019 and SCRs, we first of all consider the relevance of the adoption and application of UN legislation in certain time intervals, as well as the scientific approach to the legal language in the scientific literature indexed by Google books between 1945 and up to the present.

In a generalist approach, given the complexity and multitude of SCRs adopted in the eighty years under analysis, we examine the correlation of the most relevant resolutions for the theme selected within each figure.

Thus, the increased frequencies recorded by Figures 1, 2 for the concepts operationalized during the 1990–2000 period indicate a central theme of SCRs, namely “humanitarian” and “humanitarian assistance.” The determination of the most important associations within n-grams focused on the human factor as a basic element in the selection of adjacent words.

In this context, linguistic trends and policy outcomes have centered the increased interest in words, word associations and phrases related to the “human” topic.

From here the essence of research and analysis of international politics related and combined various associations from the lexical field of the topic “human” such as “humanitarian + noun,” namely: “humanitarian assistance,” “humanitarian aid,” “humanitarian relief,” “humanitarian grounds,” “humanitarian reasons” and “humanitarian purposes,” “humanitarian organizations,” “humanitarian need,” “humanitarian personnel” and “humanitarian crisis.”

In a second perspective, it is important to specify that these heuristic approaches in the sphere of the lexical field of the “humanitarian” associated topic allowed a wider sampling of selected n-grams offering the possibility of calibration to UNSC resolutions.

Moreover, the selection and sampling distribution of n-grams in the sphere of the lexical field of the “humanitarian” topic has associated various linguistic correlations.

From a sociological and terminological-conceptual point of view, SCRs operationalize a terminology and a distinct field focused on humanitarian causes and the spectrum of humanitarian concepts. We also find that the relatively high frequencies concretize a historical trend of SCRs evident in the last thirty years, namely the directional character of SCRs for examining and relating concepts from the semantic sphere of humanitarian causes.

A more in-depth investigation highlighted by Figure 1 reveals the increasing frequency of use of the “human rights” topic starting with the 1970s, with a more pronounced emphasis in the 1980s and very high frequencies in the 1990–2000s. This trend of increased use of the “human rights” topic expresses the semantics of the concept, i.e., the relationship between the concrete legal meaning and its external referent, i.e., the receptivity of the scientific literature as a result of the directional character generated by the adoption of some SCRs in the field.

Two relevant resolutions empirically validate this evidence: (i) S/RES/2217 (United Nations Security Council, 2015a) regarding the promotion and protection of human rights; (ii) S/RES/1265 (United Nations Security Council, 1999) on humanitarian assistance and human rights.

Figures 3, 4 engage a discussion regarding the substitutive character of SCRs, that is, the message of the resolutions refers to and aims at a recurring implementation in time and space. In Figure 3, it is noted the dynamic frequency associated with an increasing trend after the year 2000 of the use of the two topics “security environment” and “conflict prevention”.

Figure 4 highlights a double articulation in the sphere of use of two other concepts “armed conflict” and “ceasefire” with accentuated and sustained and increased trends in the frequency of use both in the space of scientific literature, but also in SCRs, a fact that proves the reflexivity of the resolutions towards the social space and the peaceful resolution of conflicts, but also a tendency towards the autonomy of the decision-making space centered on guaranteeing peace and security and conflict prevention actions.

Using the approach of historical comparativism, we note two resolutions adopted during the 2000s that empirically address the topics centered on Figures 3, 4 as follows: (i) S/RES/2225 (United Nations Security Council, 2015b) regarding violations of international humanitarian law applicable to children, as well as the mechanisms and tools for monitoring armed conflicts; (ii) S/RES/2427 (United Nations Security Council, 2018b) a resolution adopted unanimously by the UNSC and which commits and centers the protection of international rights applicable to children and the forms of humanitarian assistance and access in armed conflicts.

International peace and security have kept their valences and traditional usage unchanged in the linguistic and legal sphere in the decisive resolutions of the UNSC adopted in the last eight decades, even if the geopolitical focus has changed permanently, and the social, political and historical realities have engaged new terminological and conceptual approaches.

However, other concepts have not stopped progressing and evolving, constantly achieving the positions of pilot-topics (e.g., “international community” and “conflict resolution” in Figure 5). These concepts became, starting with the end of the 1990s and more pronounced starting with the 2000s, concepts providing new types of approaches in the decision-making and implementation process provided by the SCRs adopted in the same period.

A particular status is reserved for the concept of “international peace” with a progressive downward trend in specialized scientific literature indexed by Google books starting with the year 1950 and more pronounced in the last ten years until present.

However, the other concepts included in the analysis provided by Figures 5, 6 have constantly distributed during the period under analysis contents associated with a new inventory of concepts associating pre-existing terms frequently encountered in the specialized literature (e.g., in Figure 5—“protection of civilians,” “post-conflict situation,” “conflict resolution” and in Figure 6—topics associated with the linguistic multivalent of the “peace” concept, namely “inclusive peace,” “durable peace,” “sustainable peace,” “peace operations,” “peace agreement”).

The formulation of this opinion is based on five SCRs adopted in the sphere of security and peace: (i) S/RES/2419 (United Nations Security Council, 2018a) focused on the role of youth participation and the role of civil society in the peace process; (ii) S/RES/1963 (United Nations Security Council, 2010) which focuses on a systemic structure to guarantee human rights and collaboration as a requirement for ensuring peace and security; (iii) S/RES/1265 (United Nations Security Council, 1999)—the first resolution addressing the issue of the protection of civilians in the event of armed conflicts, calling for compliance with the legal provisions regarding international humanitarian law; (iv) S/RES/1208 (United Nations Security Council, 1998a) which affirms the responsibility of host states in the matter of hosting refugees and ensuring security and humanitarian access to refugee camps and settlements; (v) S/RES/80 (United Nations Security Council, 1950) on India-Pakistan issue focusing on mutual responsibilities and implementation of the demilitarization proposal.

The creation of a terminology centered on the relationship between the five fundamental concepts, namely “state,” “governance,” “society,” “civil society,” “resources” requires rigor and scientific objectivity generated by the new resolutions adopted by the UNSC in the period 1970–2000.

Therefore, Figure 7 concretizes a particular aspect focused on the period of the 2000s, which marks, on the one hand, the progressive upward trend of the topic of “good governance,” but at the same time, the same year marks the trend of decreasing frequency of occurrences for the topic of “power sharing.” Nonetheless, we are therefore dealing with a linguistic field focused on the prescriptive approach of the state-society relationship (Figure 8) which reveals a priority orientation both of SCRs, but also of the scientific literature starting with the 2000s focusing on the identification of theoretical and practical solutions for conflict and post-conflict situations.

More in depth, Figure 9 lists the frequency of occurrences for topics associated with the lexical field of the “public” concept (e.g., “public procurement,” “peaceful political competition,” “public funds,” and “public financial management”). This perspective with a high degree of contextualization postulates the relevance of internal factors and the designation ratio between the assistance of international financial institutions and economic rehabilitation and reconstruction.

Extending such an observation, our analysis associates the adoption in the same period of four SCRs intended to establish the role of the state in social functioning and maintaining peace: (i) Resolution S/RES/1502 (United Nations Security Council, 2003) focuses on the role of states and governance in ensuring and protecting of UN staff and associated personnel in accordance with the obligations stipulated by international humanitarian law; (ii) Resolution S/RES/1315 (United Nations Security Council, 2000) bringing a consistent contribution to the normative spectrum of peace processes (e.g., Sierra Leone), the role of the state, institutional governance and the rule of law; (iii) Resolution S/RES/1168 (United Nations Security Council, 1998b) regarding the conflict in the former Yugoslavia and the need for a judicial and police reform in Bosnia and Herzegovina in direct relation to the role of the state and the security capabilities and (iv) Resolution S/RES/1212 (United Nations Security Council, 1998c) on UN technical assistance and economic, social and institutional resilience in Haiti.

The semantics of the three central concepts of our analysis “security,” “democracy” and “human rights,” thus circumscribes, starting with the creation of the United Nations in 1945, a very broad field of research that operates in various spectrums of the social and political spectrum: security studies, political sciences, sociology of relations international law, international humanitarian law, etc.

In other words, the status of the meanings of these concepts operated according to rigorous double-conditioned criteria: (i) first of all, the linguistic variation of the concepts selected for analysis designated new realities and political, social and historical conditions generated by the evolution of the international security environment after 1945 until now, a reality transposed in SRCs adopted in the same period of time, and (ii) secondly, the selected topics circumscribed the variable side of acceptance and receptivity in the specialized scientific literature monitored in the Results section of this study.

This double lexicological conditionality also requires a systemic and functional approach to the three concepts: “security,” “democracy” and “human rights.” Thus, Figures 10, 11 investigate related topics in the descriptive-normative sphere of civil and social rights, as well as the process of regulating the legal status of vulnerable categories in the case of armed conflicts. The two figures reveal the clear observation of the appearance and evolution of the frequencies of the selected topics by mapping two referential time intervals: (i) the period of the 1980s-1990s which shows particularly high frequencies for two concepts “refugees” and “ethnic groups” (Figure 10) and (ii) the period from the 2000s to the present, which reveals constantly increased frequencies of the four topics represented in Figure 11, namely “marginalized groups,” “vulnerable populations,” “host communities” and “faith groups”.

It is appropriate to highlight the strong impact of the topics in Figures 10, 11 with corresponding normative status at the level of SCRs resolutions that give the social and psychosocial factor a special status. In this context, we note the adoption during the 2000s of three SCRs intended to formulate the strategic importance of the social factor and human behavior that autonomizes a bridge between the three fundamental concepts of the current analysis.

A first resolution in the field is S/RES/2171 (United Nations Security Council, 2014) regarding the prevention of conflicts and the maintenance of international peace and security. The second resolution S/RES/2282 (United Nations Security Council, 2016) broadens the scope of scientific interest regarding the dynamics of social relations and the role of peace sustainability, as a central aspect of sustainability and security, good governance and democracy. Therefore, the language of the scientific community also diversifies and reorients the decision-making process towards the role of negotiation and communication as a means of preventing disinformation, but also towards the guarantee of human rights, the right to free expression, the promotion of tolerance and peaceful coexistence, including by focusing on the role of cultural heritage and of interreligious and intercultural dialogue, promoting integration and social cohesion.

This paradigm shift in SCRs adopted after the year 2000 emphasizes a diversity of topics from female leadership, civil society and social cohesion, sustainable peace, freedom of expression to related forms of social mobility, guaranteeing and supporting missions to maintain peace, resources and financial mechanisms.

However, after 1950 the social, linguistic and ethno-cultural contacts, but especially the inter-linguistic interferences at the level of the community of the member states of the United Nations developed and at the same time systematized the field of scientific knowledge assisting a precise delimitation and an ascent of topics from the social sphere in within the framework of SCRs, but also at the level of scientific literature indexed by Google books starting with the second half of the last century.

In this discussion, we can add the fact that the progressively increased frequency of the occurrences of the selected topics both in SCRs and in the scientific literature is evident starting with the period of 1950–1960, being directly dependent on the emergence of a new scientific discipline, namely sociolinguistics (Tuten and Tejedo-Herrero, 2011; McConnell, 2017). Sociolinguistics focuses on the variations in the linguistic use of concepts conditioned by the social plan by introducing new aspects of the examination of selected social concepts (Holmes, 2013).

For our research, the emergence and evolution of sociolinguistics led to the progressively increased evolutions in the second half of the last century of linguistic variations in the field of sociology of international relations, but also a growing popularity in specialized literature of topics from the lexical field of human behavior (e.g., “refugee,” “ethnic groups” and “human rights violations” in Figure 10 and “marginalized groups,” “vulnerable populations,” “host communities” and “faith groups” in Figure 11).

This sociolinguistic co-variation, developed mainly after 1960, was joined by the evolution of ethno-linguistics, which led to constantly increased evolutions of topics that trace social invariants (e.g., “ethnic groups” in Figure 10 and “marginalized groups,” “vulnerable populations” and “host communities” in Figure 11).

This social dialectology answers the need for a relationship between the frequency of occurrence of the selected topics, the international security environment and the normative framework of the resolutions adopted by the UNSC.

Moreover, the importance of these terminological relationships is also based on the analysis pole of topics from the spectrum of vulnerable categories (e.g., the topics in Figure 12, namely “children,” “youth” and “women”). These three topics with autonomous status in Figure 12 strengthen the perspective of interdisciplinarity within the research between the linguistic frequencies of appearance in specialized literature and the social structure and guarantee of security and personal rights and freedoms.

This impulse was triggered at the end of the 1980s and the beginning of the 1990s when we witnessed an exponential diversification of works and researches in the specialized literature that reflected an accelerated and growing co-variation of three concepts from Figure 12 (“children’s rights,” “involvement of women” and “youth engagement”).

The analysis of the frequency of occurrences for the topics “protection for women and children,” “protection of victims,” “abuses of human rights” proves in Figure 13 the principle of linearity, in the sense that systemic variations develop progressively, especially starting with the 1980s on the other hand, the two topics “human rights violations” and “asylum-seekers” occupy a central place in Figure 13, using frequencies of maximum intensity starting with the 1970s until the beginning of the 2000s. An absolutely distinct position in the evolution of the frequency of occurrences for the topic “human rights violations” is outlined at the end of the 1990s and the beginning of the 2000s in a complex interdependence with two resolutions adopted by the UNSC in the same period (Figure 10).

The first resolution S/RES/1998 (United Nations Security Council, 2011) reflects and leads to new perspectives for arguing the normative framework established and adopted by the UNSC. The structure reflects the particular tendency to approach the relationship between citizen, state, society and rights as terms of reference for the experience of children in armed conflicts. This resolution enshrines a sociological and socio-political invoice approach reflecting an obvious concern of the UN for a more detailed circumscription for the civil character and international protection of children, durable peace and humanitarian assistance, the security and the humanitarian character of the reconstruction programmes.

In general terms, the second resolution, S/1998/1625 (United Nations Security Council, 2005), has an increased relevance for the democratic framework, for the international security environment and for the respect of human rights. The starting point of this resolution is the social and political context of the humanitarian assistance granted to refugees and other categories in conflict situations, but especially the relationship with the social spectrum and the humanitarian norms applicable to some vulnerable categories. At the same time, Figure 13 emphasizes the social dimension of human rights and develops a correlative analysis in relation to two periods, namely before and after the year 2000. Developing this chronological-normative perspective, we can highlight a sense of cohesion between the scientific literature and the legal framework of SCRs.

Moreover, trying to summarize the taxonomy of factors and institutional governance, Figure 14 reveals the variations in the frequency of topics in relation to five concepts: “political transition,” “transitional justice,” “transitional institutions,” “transitional reforms,” “transitional government.” The graphic representation of Figure 14 confirms a synchronous trend of accelerated increase in the frequencies recorded by the “transitional justice” topic. This exhaustive perspective of the “transitional justice” topic is reflected in S/2004/616 (United Nations Security Council, 2004), which theoretically and applied the “transitional justice” and “rule of law” topics, focusing on the specificities of societies in transition, but also post-conflict societies.

Moving the focus from the social behavior to the institutional governance in the last figure of the analysis (Figure 15) represents an important contribution for the perspective of the evolution of the selected subjects. Therefore, Figure 15 represents the foundation of the analysis of the institutional spectrum showing primary interest for the active role of political dialogue, judicial and institutional reform, but especially for the configuration of a continuum of interdependencies at the level of security reform starting with 1945.

In a strict acceptance focused on the six topics mapped in Figure 15, we detail in a first observation the increasing progressive and evolutionary trends for all six topics in the period 1945–2000. This projection identifies the exceptional character and essential role of political dialogue and legal and institutional reform for the institutional governmental space. The second observation deals with the context of the synchronous evolution of the tips in the sphere of political dialogue and reform, which permanently validates the status both in the scientific community, but also in the UNSC of creation, re-creation, re-configuration and 0regulation of the social, political and security realities of the last eighty years.

A significant contribution to this issue is the space of convergence between five resolutions adopted by the UNSC in the period 1995–2017, as follows: (i) S/RES/146 (United Nations Security Council, 1960a), S/RES/151 (United Nations Security Council, 1960b) and S/RES/152 (United Nations Security Council, 1960c) and S/RES/1040 (United Nations Security Council, 1996a) referring to the role of political dialogue, reconciliation and security in Gabon, Congo and Burundi; (ii) S/RES/858 (United Nations Security Council, 1993), S/RES/937 (United Nations Security Council, 1994) and S/RES/1077 (United Nations Security Council, 1996b) establishing an office under the auspices of the United Nations Observer Mission in Georgia and extending its mission; (iii) S/RES/1031 (United Nations Security Council, 1995) regarding a political agreement negotiated within the framework of the conflicts in the former Yugoslavia and the implementation of the Peace Agreement for Bosnia and Herzegovina; (iv) S/RES/2388 (United Nations Security Council, 2017) regarding human trafficking in the areas affected by armed conflicts, but also in post-conflict areas and S/RES/8 (United Nations Security Council, 1946) on new admissions of Member States to the UN and (v) S/RES/505 (United Nations Security Council, 1982) on Falkland Islands (Malvinas) aimed to restore dialogue, reconciliation and peace.

The five resolutions form a unitary whole, of dependence and interdependence between the need for security, the desires of democracy and the protection of human rights. Moreover, the resolutions mentioned above find their original essence in the sequence of historical, social and political development and evolution of the international environment, but also in the particular experience of the areas affected by the conflict, as well as in the post-conflict period.

Thus, the conceptualizations and discussions related to the five figures (Figures 1–15) are necessary and indispensable for the monitoring of the selected subjects, generating a framework of resonance and complementarities between the decision-making space, through the analysis of the relevant resolutions in the field adopted by the UNSC, and the space the scientific community represented by specialized literature indexed and listed by Google book. Secondly, this dialectic defines and particularizes the participation of the international community by designating the formative principle of the UNSC resolutions, but also the systematic nature and conceptual-terminological value developed by specialized literature in the period 1945–2019.

### 4.1 Practical implications of the study and relevance for humanitarian interventions and peacebuilding

The practical implications of the study allow a two-steps analysis. First of all, the graphs of ngrams variations in the selected period and the results obtained allow the identification of frequency variations, but also the easy identification of some periods that require an additional deepening by pointing to the causalities, but also the inputs and outputs that change the limit of the values obtained. On the other hand, the frequency variations and the monitoring of selected topics and word associations generate a typical analysis matrix of both UNSC resolutions and the scientific literature that develop useful tools for forecasting and developing policies and strategies both in the decision-making sphere, but also the sphere of policy instruments in the field of security and human rights. Moreover, the present study focuses on n-grams research by focusing on two focal notions, namely humanitarian assistance and peacebuilding with the aim of responding to the concerns of the international community in this field after 1945. In this direction, the study extends the formal analysis framework to a complex of associations of words and lexical phrases associated with the two notions, humanitarian assistance and peacebuilding, and the quantifications obtained present valuable contributions for the decision-making factors, but also for humanitarian organizations, the social sector, state actors and governmental organizations. At the same time, such an approach articulates a complex monitoring of the knowledge of UNSC resolutions, but also a recognition of the role of scientific literature in contemporary society through the accumulation of relevant assessments and contributions in the field of human security, contemporary humanitarianism and humanitarian action. Consequently, the digitization and quantification of n-grams reflects the applicability of decisions adopted by the UNSC that are essential for global security and humanitarian assistance.

## 5 Conclusion

The results of the research expanded the field of reference and analysis of the three notions of security, democracy and human rights by integrating the multifactorial and multi-conditional analysis for the interpretation of the results of the fifteen figures and the compliance to the SCRs adopted beginning with 1945 till 2019. This section of the research answers the first research question (Q1) by outlining the context when the conceptual trends related to security, democracy and human rights appear and evolve in the resolutions of the UN Security Council (UNSC) during the selected analysis period of 1945–2019?

To conclude, we can highlight the following:

1. The results of the frequency analysis through the online monitoring arranged with the help of Ngrams have generated an overview of the thematic clusters of the research in the sphere of international politics topics, showing the progressively increasing developments in the specialized literature since 1945 till 2019. Consequently, the results obtained throughout this stage of the analysis answers the second research question (Q2) which addresses the manner in which the security-democracy-human rights relationship evolve in the scientific literature. Moreover, it also addresses the third research question (Q3) regarding the frequency of appearance in the online indexed scientific literature from the period 1945–2019 of selected concepts and word associations (n-grams) specific to the security-democracy-human rights relationship.
2. Being a comparative exploratory research, the results of the analysis of ngrams were permanently compared and systematically analyzed with the main resolutions of the UNSC adopted in the same period. Thus, the research detaches itself from the subjectivity, the vision and the often contingent interests of the international actors and adopts the vision of the UNSC resolutions quasi-majority accepted in the last eight decades. Hence, it reciprocates to the fourth research question (Q4) which aims at identifying whether there is a correlation between UN Security Council (UNSC) resolutions and the frequency of appearance of concepts in the scientific literature during 1945–2019. The paper draws its insights from the analysis of 25 UN Security Council (UNSC) resolutions on conflict and conflict resolution adopted between 1946 and 2019 during a 72 year time-span.
3. The results mapped in the fifteen figures reveal recent answers regarding the evolution of politics and the sociology of international relations, integrating concepts and associations of “border” topics that have interfered with the research scope of specialized literature, a fact that has assumed new linguistic disciplines such as sociolinguistics and ethnolinguistics. These facts give the research results distinction and applicative validation. Thus, the results acknowledge the fifth research question (Q5) on determining whether the concepts and terminology operationalized in the UN Security Council (UNSC) resolutions during a certain period indicate a central theme in the scientific discourse.
4. Deriving from the hermeneutic approach of the UNSC resolutions, the results show the diversity of inter and intradisciplinary perspectives that contribute to an understanding and interpretation of the selected topics in the global sphere. In other words, correlating the results of online monitoring using the Ngram tool with the normative and argumentative knowledge of UNSC resolutions generates a valuable investigation of a normatively exploratory and linguistically descriptive nature. Therefore, the topics selected are common concepts of the lexicon of the sociology of international relations and the use of terminology in the fifteen thematic figures is precise and satisfy the aims expressed through the sixth research question (Q6) referring to the capacity of selected concepts and terminology to coin new types of approaches in the decision-making and implementation process of the UN Security Council (UNSC) resolutions. Bearing these assumptions in mind, the research is future-driven as it wonders on the manner in which concepts and terminology coin new types of approaches in the decision-making and implementation process in additional fields and areas specific to the security-democracy-human rights relationship and challenges such as climate change, food security and the access to vital water resources, democracy and cybersecurity. Aiming at overrunning the limitations of the present study, future research should benefit from the availability of online data once Google Ngram Viewer platform succeeds in covering and providing access to post-2020 digitalized scientific literature. The accessibility of post-2020 digitalized academic resources is a vector for research in the field of human rights, elections and democracy issues, health, the sanitary crisis and human security, economic security, recovery and resilience, global energy crisis, climate change, regional and international security developments.
5. In addition, through the reflected position regarding the relationship between the frequencies of online monitoring and the reality of the lexicon of resolutions adopted by the UNSC, the research inaugurates a new thematic direction for future investigations regarding the interactions between the specialized research agenda and the terminological precision of the decision-making spectrum of the institutional governance at international level.

An important aspect of the study focused on research ethics and the integrity of the data accessed and processed during the research. Considering that the examination of data and information involved the tools provided by Google Ngrams and UNSC, we developed a framework for mapping the selected data piloting on the one hand a meta-analysis of twenty-five resolutions and a complex screening of scientific literature published over seven decades. Based on a preliminary analysis, we selected and validated in the first stage UNSC resolutions relevant to the institutional governance and the implementation process. In the second stage, we correlated and concentrated on the defining aspects related to the contribution of the scientific literature, guaranteeing the reliability and importance of the research. The answers identified by the research describe the mechanisms of international governance, reveal the impact of social phenomena, as well as the consequences of political decisions. This approach contributes to the understanding and deepening of an improved management of the field of knowledge both for the academic and non-academic sectors.

Considering all the above, we appreciate that the diversity of the current study proves that there is a complementarity of the research directions of the topics in the sphere of international relations at the border between the spectrum of specialized online monitoring and the political and social reality, i.e., between what is communicated and signified and what is significant, written and decisional.

The research has nuanced the evolution and transformations at the nexus of international politics, international relations, sociolinguistics and ethno-linguistics as complementary disciplines by exploring the variations in the frequency of appearances of the selected concepts in the specialized literature within an integrated analysis of contextual, historical, political, institutional, legal and social factors institutionally ascertained in the resolutions of the Security Council (SCRs) of the United Nations. The exploratory discourse analysis has highlighted the birth of concepts and conceptual associations and the dynamics frequency within discursive use.

## Data Availability

The original contributions presented in the study are included in the article/supplementary material, further inquiries can be directed to the corresponding author.
